# Early *Poplar* (*Populus*) Leaf-Based Disease Detection through Computer Vision, YOLOv8, and Contrast Stretching Technique

**DOI:** 10.3390/s24165200

**Published:** 2024-08-11

**Authors:** Furkat Bolikulov, Akmalbek Abdusalomov, Rashid Nasimov, Farkhod Akhmedov, Young-Im Cho

**Affiliations:** 1 Department of Computer Engineering, Gachon University, Sujeong-Gu, Seongnam-si 461-701, Republic of Korea; bolikulovfurkat@mail.ru (F.B.); a.abdusalomov@tsue.uz (A.A.); 2 Department of Information Systems and Technologies, Tashkent State University of Economics, Tashkent 100066, Uzbekistan; rashid.nasimov@tsue.uz

**Keywords:** “Poplar-Disease” dataset, deep learning, contrast stretching, YOLOv8, *Poplar* (*Populus*) disease detection, object detection

## Abstract

*Poplar* (*Populus*) trees play a vital role in various industries and in environmental sustainability. They are widely used for paper production, timber, and as windbreaks, in addition to their significant contributions to carbon sequestration. Given their economic and ecological importance, effective disease management is essential. Convolutional Neural Networks (CNNs), particularly adept at processing visual information, are crucial for the accurate detection and classification of plant diseases. This study introduces a novel dataset of manually collected images of diseased *poplar* leaves from Uzbekistan and South Korea, enhancing the geographic diversity and application of the dataset. The disease classes consist of “Parsha (Scab)”, “Brown-spotting”, “White-Gray spotting”, and “Rust”, reflecting common afflictions in these regions. This dataset will be made publicly available to support ongoing research efforts. Employing the advanced YOLOv8 model, a state-of-the-art CNN architecture, we applied a Contrast Stretching technique prior to model training in order to enhance disease detection accuracy. This approach not only improves the model’s diagnostic capabilities but also offers a scalable tool for monitoring and treating *poplar* diseases, thereby supporting the health and sustainability of these critical resources. This dataset, to our knowledge, will be the first of its kind to be publicly available, offering a valuable resource for researchers and practitioners worldwide.

## 1. Introduction

*Populus* nigra, or black *poplar*, is a critical species in global riparian forest ecosystems. Historically, black *poplar* was abundant across Europe, but its numbers have dramatically decreased due to human activities such as river management, diseases, and wood cutting. This reduction is alarming because black *poplar* plays a vital role not only ecologically—contributing to the biodiversity and stability of river habitats—but also economically, as it is used in soil protection and reforestation efforts in polluted areas [1]. The planting material of poplar is of great significance in raising plantations. Only source-identified planting material should be used. This is because *poplars* are raised clonally, and there are several thousand clones in cultivation. They are site-specific and locality-specific. Their wood density is also variable. They are specific to a particular day, length of light, and temperature. It is not easy to identify a clone at the nursery stage unless one inspects them daily and records their characteristics [2]. From an economic perspective, *poplars* are used in the timber industry for products like paper and plywood due to their rapid growth and regeneration capabilities. They are also used in bioenergy production and phytoremediation projects due to their ability to accumulate heavy metals and other contaminants, helping to clean up polluted soils and water. Moreover, studies on *poplars* have helped in explaining the storage and mobilization of nutrients within trees, offering insights into tree physiology that are crucial for forestry management and conservation efforts. The ability of *poplars* to adapt to different environments and their role in carbon sequestration further underline their environmental importance [3]. *Poplars* are susceptible to various diseases and pests that can decimate populations, especially when trees are stressed by other environmental factors. Reduced genetic diversity is caused by human activities like selective logging and habitat fragmentation, which can lead to reduced genetic diversity in *poplar* populations, making them more vulnerable to diseases, pests, and environmental changes [4]. Detecting these diseases early is crucial for their preservation. There are many methods to detect the diseases in trees, for example, visual inspection, laboratory testing, remote sensing, and aerial imaging. Identifying symptoms through expert analysis is both time-intensive and frequently too delayed to allow for effective treatment [5]. Regular monitoring and surveillance of *poplar* stands are crucial for the early detection of disease, pest infestation, or environmental stress. Early detection allows for timely interventions and can help prevent the spread of pathogens. Maintaining and enhancing genetic diversity within *poplar* populations also play a vital role in increasing their resilience to pests and diseases. This can be achieved by planting a mix of genetically diverse individuals that are resistant to various diseases and environmental stresses. Sanitation and hygiene practices such as removing diseased and dead trees and cleaning pruning tools can minimize the spread of pathogens. Infected plant materials should be disposed of safely and away from healthy trees. In cases where it is necessary, the use of fungicides and pesticides can control the spread of certain diseases and pests, although these should be used judiciously to minimize environmental impacts and avoid the development of resistance. Biological controls, such as introducing natural predators or beneficial microorganisms that combat pests and diseases, offer an environmentally friendly alternative to chemical treatments. Implementing quarantine measures and restricting the movement of plant materials from areas known to be infested can prevent the introduction and spread of pathogens and pests to new areas. Furthermore, developing disease-resistant poplar varieties through traditional breeding or genetic engineering provides a long-term sustainable solution. This process involves selecting traits that enhance resistance to specific diseases and environmental stresses. Educating and training forestry staff and stakeholders on the identification of diseases, appropriate treatment methods, and management practices can also greatly enhance the effectiveness of disease control strategies [6].

Deep learning (DL) and Convolutional Neural Networks (CNNs) have been extensively utilized in recent research [7]. They offer a revolutionary approach to identifying and diagnosing these diseases early on, thereby allowing for timely intervention and treatment [8]. By training models on images of *poplar* leaves, branches, and bark exhibiting signs of infection, these algorithms can learn to recognize the subtle nuances that differentiate healthy from diseased trees. One of the key advantages of using CNN architectures in disease detection is their ability to process and analyze complex image data, identifying patterns and features that may not be visible to the human eye [9]. This capability is particularly beneficial for detecting diseases that manifest through minor changes in leaf color, texture, or pattern. There has been significant research conducted to detect plant diseases, with a focus on *poplar* diseases. A review of the existing literature underscores a significant hurdle in progressing research within this domain: the scarcity of natural or real-world datasets for *poplar* diseases. Furthermore, existing real-world datasets that could potentially be useful are privately held and not available to the broader research community.

Our preliminary research efforts focused on identifying diseases affecting *Poplar* (*Populus*) trees revealed that the predominant issues include leaf miner infections, spotted wilt virus, and nutritional deficiencies. As a result of the studies, we found that the most important *poplar* leaf diseases are those mentioned above, “Parsha (Scab)”, “Brown-spotting”, “White-Gray spotting”, and “Rust”. Regrettably, there are no publicly accessible datasets that encompass these specific disease categories, and we also noted instances where a single leaf exhibited multiple disease afflictions. To mitigate these challenges and advance research in this field, we propose the development of a novel dataset named “Poplar-Disease”. This initiative aims to be the first to offer a publicly available dataset that includes the multilabelling technique, object detection data specifically targeting real-world cases of *poplar* trees affected by leaf miner, spotted wilt virus, and nutritional deficiencies. In conjunction with this new dataset, we also implemented a comprehensive *poplar* disease detection system designed to operate effectively in real-world environments. This system and the accompanying dataset are poised to enhance diagnostic accuracy and facilitate more effective management of *Poplar* (*Populus*) diseases.

**Figure 1:**
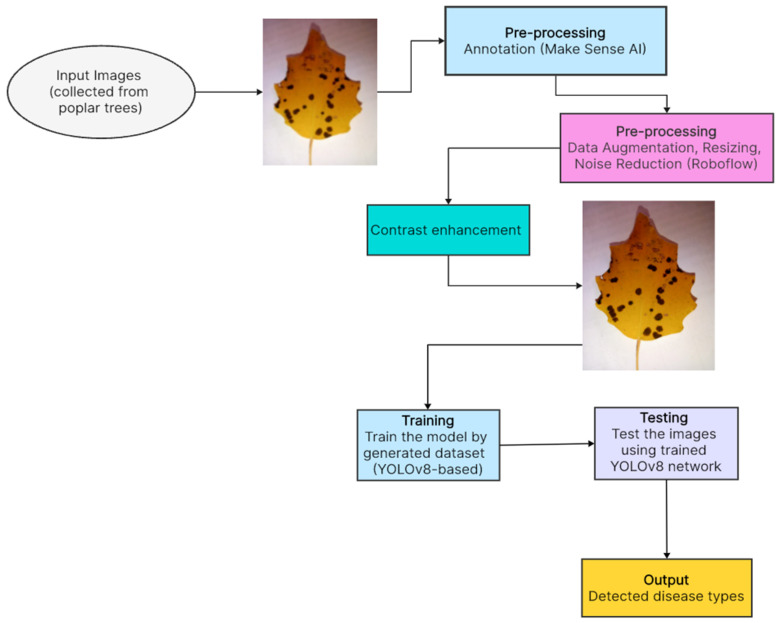
Description of our proposed method.

Our study introduces, as described in Figure 1, significant advancements in *poplar* disease detection through the development of a YOLOv8 model, supported by a newly compiled dataset. The key contributions include the following:

1. Data Collection: Gathering a diverse and comprehensive collection of images featuring *poplar* trees affected by various diseases. This involved extensive fieldwork and the compilation of images from various sources to create a robust dataset for analysis.
2. Expert Classification: The images were expertly classified and labeled, ensuring the dataset’s accuracy and reliability for model training.
3. Generating New Poplar Disease-Based Dataset: Using the collected images to create a new dataset specifically focused on *poplar* diseases. This dataset includes labeled images where each image is annotated with information about the specific diseases affecting the *poplar* trees depicted.
4. Powerful Model Training (YOLO): Utilizing state-of-the-art deep learning techniques, such as the YOLO (You Only Look Once) model, for training a powerful machine learning model. YOLO is known for its speed and accuracy in object detection tasks, making it well suited for identifying and localizing diseases in images of *poplar* trees leaves.

These contributions aim to facilitate early disease detection and support ongoing research, displaying our commitment to improving plant health monitoring. The structure of this paper on *poplar* disease detection is methodically organized to facilitate understanding and exploration of the topic, as follows: Section 2 delves into a review of existing studies, focusing on the methods traditionally employed for identifying specific properties and indicators of *poplar* diseases. In Section 3, we introduce our proposed approach to *poplar* disease detection, detailing the methodology, the development of the YOLOv8 model, and the compilation of a comprehensive dataset. Section 4 presents the results of our experiments conducted using the newly assembled database, providing a thorough analysis and discussion of the findings. Section 5 addresses the limitations and future work encountered with the proposed method, offering insights into the challenges and potential areas for refinement. Concluding the paper, Section 6 summarizes our main contributions and outcomes, while also outlining directions for future research to further advance the field of *poplar* disease detection.

## 2. Related Works

In the sphere of digital technologies for the automated detection of plant diseases, prevailing techniques are classified into two principal categories: traditional methods which utilize Computer Vision, and more advanced approaches that incorporate artificial intelligence (AI), specifically focusing on machine learning (ML) and DL. This narrative seeks to explore these two fundamental methodologies in depth. Nonetheless, it is important to recognize that the deployment of innovative technologies and the creation of well-curated datasets are essential for the precise and efficient detection of plant diseases.

### 2.1. Computer Vision and Image Processing Approaches for Poplar (Populus) Disease Detection

A study by Shahryar Sedighi et al. [10] explored an innovative image processing technique using a Laplacian threshold for the early detection of leaf spot disease in *poplar* trees. They utilized specific illumination wavelengths selected with a UV-Visible Spectrophotometer and a digital camera mounted on a stereomicroscope. Despite the method’s potential for accurately identifying disease presence and progression, the method’s accuracy is highly dependent on precise lighting conditions, which can vary significantly in natural environments, potentially affecting the consistency and reliability of disease detection. A study by Ming Hao et al. [11] aimed to enhance *poplar* leaf disease recognition by preprocessing images to highlight early-stage disease characteristics. They used an advanced edge detection method, adaptive histogram equalization to adjust for uneven lighting, and OTSU segmentation for lesion extraction, with these preprocessed images then analyzed using an AlexNet neural network. Despite achieving higher recognition accuracy, the method’s effectiveness heavily relies on controlled lighting conditions, pointing out a significant limitation in its practical application due to environmental variability. Xuan Liu et al. [12] proposed a novel leaf detection method combining Mask R-CNN with DBSCAN, utilizing RGB-D data for the accurate segmentation of overlapped poplar seedling leaves under heavy metal stress. Although encoding depth information with a jet colormap is effective for enhancing detection rates, this approach, along with the necessity for camera parameter-based conversion of depth information into 3D point clouds, introduces additional preprocessing complexity. Dashuang Liang et al. [13] introduced an enhanced CenterNet (ECenterNet) model for detecting diseased plants using images captured by Unmanned Aerial Vehicles (UAVs). This model showed significant improvements in accuracy over the original CenterNet without increasing the run time or model size. It incorporated strategies, such as Separating Overlapping Center Points (SOCP), Controlled Sampling Strategy (CSS), and a Positive Pixel Choosing Mechanism (PPCM), which collectively improved the detection accuracy by better handling object overlap, ensuring accurate bounding box recalculations for objects on image boundaries and selecting the most suitable pixels for object representation, respectively. Despite these advancements, we noted challenges in detecting infected plants due to the subtle color differences among them, contrasting with the higher accuracy in detecting dead plants, which are characterized by more uniform colors. The improvements brought about by SOCP were minor compared to CSS and PPCM, likely because the test set contained fewer instances of objects with overlapping centers due to the size of the UAV images. The research highlighted the potential for further optimization of the model, suggesting that future work could explore the precise tuning of the newly introduced hyperparameters using Neural Architecture Search (NAS) technology, aiming for a model capable of efficient and accurate plant disease detection directly from UAV devices.

### 2.2. Deep Learning Approaches for Poplar Disease Detection

Marc J et al. [14] introduced deep learning models to investigate the epigenomic correlates of *Populus* balsamifera traits, focusing on natural DNA methylation variation. They explored the potential of using DNA methylation patterns to estimate traits of industrial significance in a genetically diverse population of balsam poplar trees. Utilizing statistical learning experiments powered by deep learning models, this study achieved transparent modeling of plant traits in novel genotypes based on a small number of methylated DNA predictors. The research demonstrated the potential of DNA methylation-based models to capture tissue-specific epigenetic mechanisms underlying plant phenotypes in natural environments and offered new insights into the epigenetic influence on plant traits. These models could serve as a strategy for validating the identity, provenance, or quality of agroforestry products, underscoring the method’s innovative approach to enhancing disease recognition accuracy, albeit with the mentioned practical application challenges in variable outdoor settings. A study by Yong Wang et al. [15] discussed recent advancements in applying DL methods to forestry, highlighting the wide-ranging utility of these approaches across various forestry-related tasks. The authors note DL’s effectiveness in sawn timber surface quality evaluation, forest resource surveys, tree species identification, wood moisture content prediction, and forestry information text classification. Despite DL’s potential to significantly improve accuracy and efficiency in these areas, this study also acknowledges the limitations and challenges associated with implementing DL in forestry. These include the complexity of DL theories for forestry researchers, the requirement for large datasets to train DL models effectively, the labor-intensive process of data labeling, and the difficulty of applying DL models trained on small or non-diverse datasets to broader real-world forestry applications. The paper concludes by emphasizing the need for further refinement of DL methods in forestry and predicts future trends in DL applications, including more extensive integration with other emerging technologies to advance smart forestry practices. Wenli Gao et al. [16] focused on the rapid estimation of holocellulose content in *poplar* clones using machine learning algorithms combined with Raman spectroscopy. They tested various machine learning models, including regularization, classical ML algorithms, and advanced gradient boosting methods, like LightGBM, CatBoost, and XGBoost. Their findings demonstrated that the advanced models, especially CatBoost and XGBoost, offered high predictive accuracy with $R^{2}$ values above 0.93 and RMSE values lower than 0.29%. This approach presents a promising tool for predicting holocellulose content in poplar, applicable in large-scale genetic and breeding programs. A study by K. Nirmaladevi et al. [17,18] introduced an innovative approach to predict leaf diseases and pest detection in agriculture using deep learning and image processing techniques. This method aims to address the significant losses in agriculture due to pests and diseases, which can affect up to 30% of crops, impacting the economic development tied to agricultural productivity. To overcome limitations, the authors propose an automatic detection system utilizing a classification algorithm inspired by the functioning of the human brain. The proposed system is designed to be cost-effective, accurate, and capable of identifying a wide range of plant diseases and pests. It represents a significant advancement over existing methods, which often rely on specific algorithms like the Nearest Neighbor or Principal Component Analysis and suffer from limitations such as slow classification with large datasets, loss of critical information during image processing, and lower accuracy. Korznikov et al. [19,20] performed individual tree recognition using a U-Net-like CNN architecture in the mixed forests of the Primorsky Region, Russian Far East, utilizing pansharpened RGB satellite images from GeoEye-1. The study parametrized the standard U-Net CNN, trained it on manually delineated images for segmentation, and compared it with traditional machine learning methods enhanced by pattern-specific features from grey-level co-occurrence matrices (GLCMs). The essence of these studies underscores the growing relevance and efficiency of deep learning techniques, particularly CNNs, in environmental and forestry applications. By leveraging very high-resolution satellite imagery and advanced neural network architectures, these approaches offer novel solutions to traditional challenges in tree species identification and forest monitoring, displaying the potential to transform ecological research and conservation strategies with high accuracy and reduced manual effort. Object detection is still one of the main challenges in the Computer Vision research area. State-of-the-art methods for object detection primarily rely on intensity-based features. Networks such as YOLO set new benchmarks in detection accuracy and speed by leveraging innovating architectural designs. Many of the advanced end-to-end trained YOLO framework-based models represent an advantage in object detection and classification [21,22,23,24,25,26,27,28,29,30].

## 3. Materials and Methods

### 3.1. Data Collection and Preprocessing

**Figure 2:**
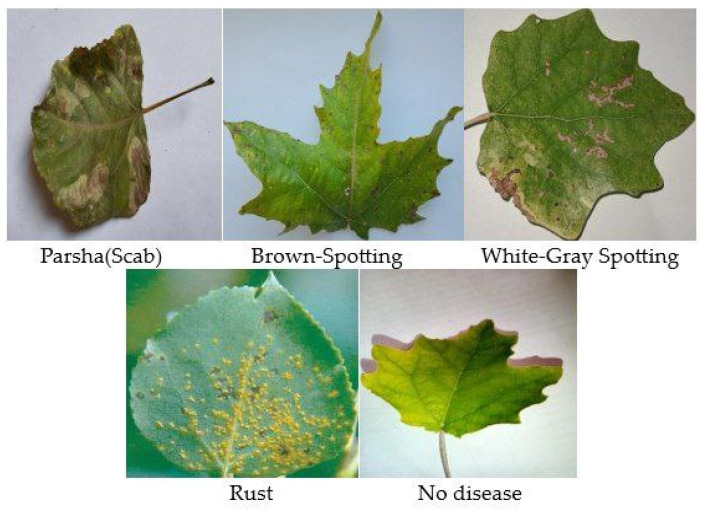
Classes of diseases on the leaves.

Undoubtedly, the most crucial aspects of training a model in AI are a high-quality dataset and a robust model. Even if we have modern models, the lack of sufficient data can pose a significant problem. This was precisely our case since we could not find a quality dataset or even images of *poplar* leaves from officially open sources. Consequently, we embarked on creating our quality dataset, understanding its potential to aid future researchers. Considering the different environments, we collected poplar leaves from three regions of Uzbekistan and South Korea, namely Samarkand, Navoiy, and Jizzakh, during the months of August and September. These months were chosen because the *poplar* leaves are sufficiently grown and can provide images clear enough for the model to detect diseases. Following the advice of our experts, we started collecting images of both diseased and healthy *poplar* leaves. For this task, we utilized the latest and most powerful digitals with cameras with 12 MP and an f/1.6 aperture of Samsung, South Korea, which can capture high-quality images. This technology ensures excellent photo clarity and detail, even in low-light conditions. We managed to collect 2357 diseased and healthy poplar leaves. After a 20-day collection period, our experts manually classified the leaves into healthy and diseased categories, resulting in 362 healthy leaves and 1995 diseased ones. With expert assistance, we began labeling the diseased leaves, ensuring each was accurately labeled. Working together, we focused on identifying four diseases, which are described in Figure 2: “Parsha (Scab)”, “Brown Spotting”, “White-Gray Spotting”, and “Rust”. See Table 1.

We used the Make Sense AI [26], choosing the polygon structure to label the diseased spots accurately. It was crucial to note that some leaves showed signs of two or more diseases, each of which was labeled separately. The annotated values were saved in JSON files that store the coordinates of the disease in the image. We downloaded our labeled data in JSON format from Make Sense AI and used Roboflow [27] for augmentation, specifically 90-degrees clockwise and counterclockwise rotations, and rotations from −15 to +15 degrees [28], employing Computer Vision techniques for this purpose. This process ensured the data, now increased to 6155 images as shown in Table 2, were in the necessary format, specifically txt, because we train YOLO with txt-formatted data.

**Figure 3:**
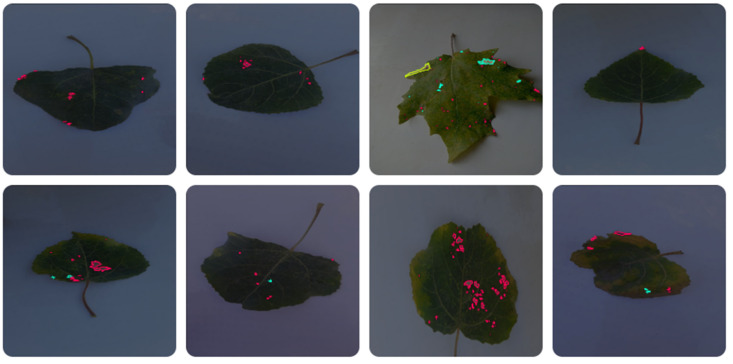
Examples of labelled *poplar* leaves before augmentation.

**Figure 4:**
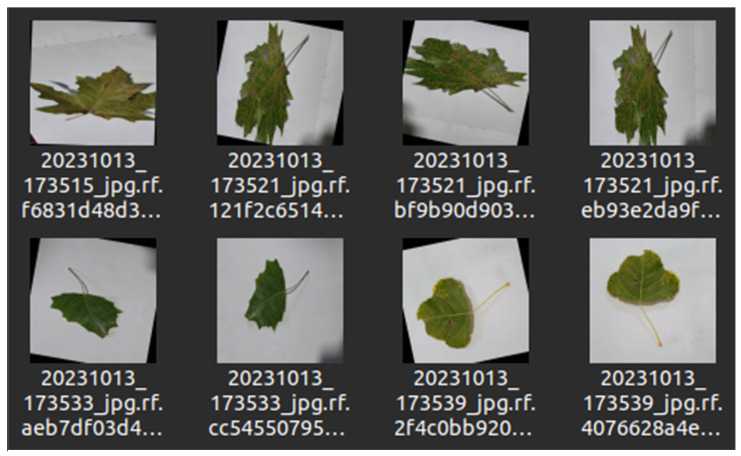
Examples of training dataset after augmentation process.

This step was vital to avoid any color alterations, as maintaining the diseased part of the leaf in its original state is crucial for accurate disease detection. After applying these technologies, the image count reached 6155. As shown in Figure 3 and Figure 4, all images were labeled by us, augmented, and increased in number to enhance the machine’s learning capability.

### 3.2. Proposed Method and Model Architecture

Our primary objective is to enhance the detection of diseases in *poplar* leaves by leveraging a sophisticated detection model that has been fine-tuned using YOLOv8 technology. The challenge of identifying diseases in plants lies in the minuscule size and often indiscernible characteristics of the affected areas on the leaves. These areas frequently blend into their surroundings due to the varied shapes and sizes they can take on, which in turn can diminish the clarity and contrast of the images captured. To solve these issues and improve the quality of the images, we employ Contrast Stretching [29], a technique that broadens the spectrum of intensity levels in images of diseased leaves, thereby making the diseased spots more distinguishable. The reliance of object detection algorithms on distinct features and patterns means that variability in the appearance of leaves and symptoms can significantly hinder the performance of traditional detection models. Moreover, the necessity for real-time processing to analyze a continuous influx of data, such as video streams, highlights the need for models that can rapidly process information without substantial delays. Sluggish processing speeds could result in a disconnect between the detection of a disease and the response to it. Given these challenges, the adoption of YOLOv8 is a strategic choice due to its efficiency and effectiveness in handling diverse aspect ratios and ensuring prompt disease detection. This approach aims to mitigate the limitations associated with conventional object detection methods and to advance the promptness and accuracy of *poplar* disease identification.

### 3.3. The Model Structure of YOLOv8 Network

YOLOv8 represents the latest advancement in the YOLO (You Only Look Once) series, setting new benchmarks in real-time object detection and classification. It is designed to process images in a single evaluation, drastically reducing the time needed for detection while maintaining high accuracy. YOLOv8 enhances its predecessors by optimizing both the speed and precision of detection, making it capable of identifying and classifying objects in complex environments with remarkable efficiency. This model integrates advanced neural network architectures and machine learning techniques to improve upon the limitations of earlier versions, offering a more robust solution for various applications in surveillance, autonomous vehicles, and beyond.

**Figure 5:**
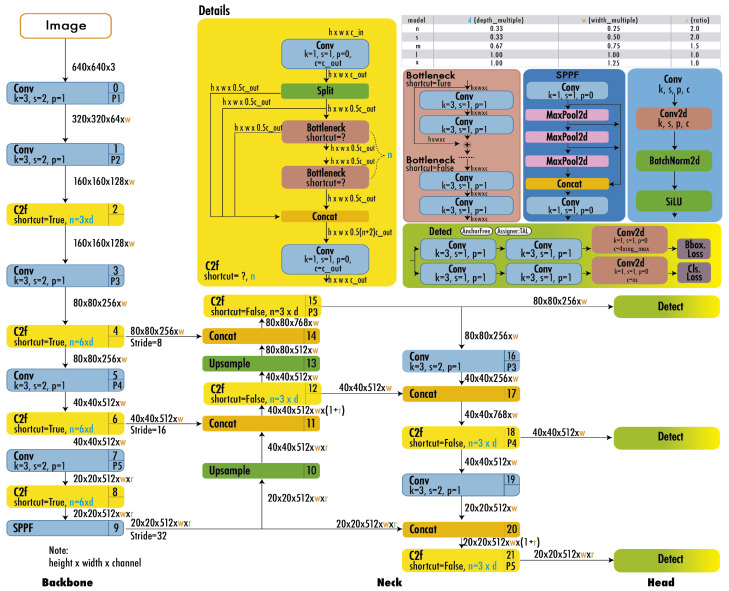
Architecture of YOLOv8 [30].

Figure 5 illustrates the YOLOv8 architecture. The architecture uses a modified CSPDarknet53 backbone. The C2f module replaces the CSPLayer used in YOLOv5. A spatial pyramid pooling fast (SPPF) layer accelerates computation by pooling features into a fixed-size map. Each convolution has batch normalization and SiLU activation. The head is decoupled to process objectness, classification, and regression tasks independently.

### 3.4. Techniques for Enhancing Image Quality

Enhancing the quality of images is crucial for various applications in photography, Computer Vision, and digital imaging. Several techniques can be used to improve image contrast, sharpness, and overall visual appeal. Here are some notable techniques:

Contrast Stretching enhances the contrast of an image by expanding the range of intensity values. This method transforms the pixel values so that the minimum and maximum intensity values of the original image are stretched to cover the full range of possible intensity values, typically from 0 to 255 for an 8-bit grayscale image. This technique helps in improving the visibility of features in the image. Histogram Equalization improves the contrast of an image by redistributing the intensity values so that the histogram of the output image is approximately flat. This method enhances the global contrast of the image and is particularly useful for images with backgrounds and foregrounds that are both bright or both dark. Adaptive Histogram Equalization (CLAHE) enhances local contrast and improves the definition of edges in each region of an image. Unlike standard histogram equalization, which works on the entire image, CLAHE operates on small regions (tiles) in the image, making it more effective for images with varying lighting conditions. Gamma Correction adjusts the brightness of an image by applying a power-law transformation. It is used to enhance both dark and bright regions of an image. By choosing an appropriate gamma value, the visibility of details in different intensity ranges can be significantly improved. Unsharp Masking enhances the edges of an image, making it appear sharper. It works by subtracting a blurred version of the image from the original image, which enhances the edges while preserving the overall smoothness of the image [31,32,33,34,35].

### 3.5. Contrast Stretching Technique

Applying the Contrast Stretching technique to enhance the visibility of *poplar* leaf images is one of the key strategies in our *poplar* disease detection study. Given that *poplar* trees are often situated in environments prone to humidity, our goal is to sharpen the accuracy in detecting *poplar* diseases according to the features of the leaves.

**Figure 6:**
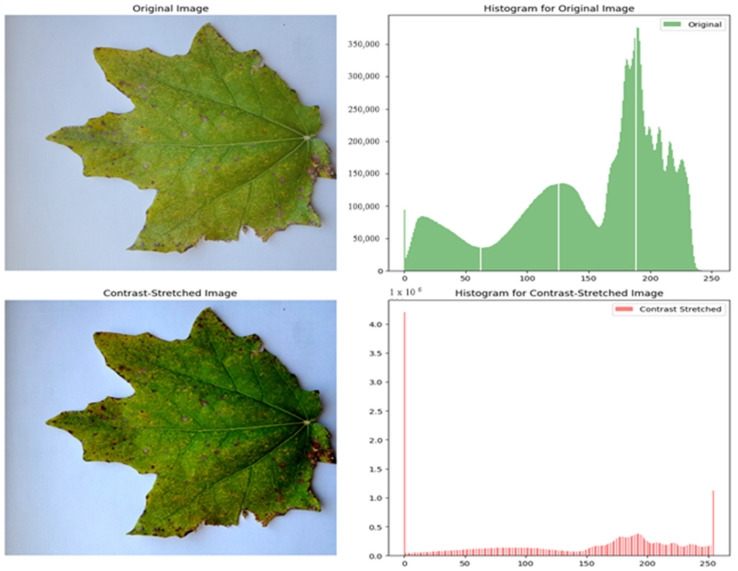
Contrast Stretching image technique.

High humidity can lead to diminished image quality, affecting the detection system’s ability to perform accurately due to issues like low visibility, image blurring, and changes in leaf surface appearance. To counteract these challenges, we incorporated the Contrast Stretching technique [36] into our *poplar* disease detection model. Before the technique can be performed, it is necessary to specify the upper- and lower-pixel value limits over which the image is to be normalized. Often, these limits will just be the minimum and maximum pixel values that the image type concerned allows. For example, for 8-bit gray-level images, the lower and upper limits might be 0 and 255. Call the lower and the upper limits *a* and *b*, respectively. As shown in Figure 6, the simplest sort of normalization then scans the image to find the lowest and highest pixel values currently present in the image. Then, each pixel *P* is scaled using the following function:

(1) $$P_{out}=(P_{in}-c)\left(\frac{b-a}{d-c}\right)+a$$

Values below 0 are set to 0, and values around 255 are set to 255.

The problem with this, as illustrated on (1) formula, is that a single outlying pixel with either a remarkably high or incredibly low value can severely affect the value of *c* or *d*, and this could lead to very unrepresentative scaling. Therefore, a more robust approach is to first take a histogram of the image and then select *c* and *d* at, say, the 5th and 95th percentile in the histogram (that is, 5% of the pixel in the histogram will have values lower than *c*, and 5% of the pixels will have values higher than *d*). This prevents outliers from affecting the scaling too much.

Another common technique for dealing with outliers is to use an intensity histogram to find the most popular intensity level in an image (i.e., the histogram peak) and then define a cutoff fraction, which is the minimum fraction of this peak magnitude below which data will be ignored. The intensity histogram is then scanned upward from 0 until the first intensity value with contents above the cutoff fraction. This defines *c*. Similarly, the intensity histogram is then scanned downward from 255 until the first intensity value with contents above the cutoff fraction. This defines *d*.

## 4. Experimental Results

This study lays solid groundwork for the successful identification of diseases on *poplar* leaves, while also highlighting the substantial promise of Computer Vision and DL techniques in tackling pivotal health issues in agriculture. Through detailed assessment using metrics such as *Precision*, *Recall*, and *F1 score*, we gain in-depth insight into the model’s efficacy, confirming its suitability for real-world application in *poplar* disease management. Moreover, the encouraging results from our initial trials prompt ongoing research and enhancement of these techniques, aiming to significantly improve *poplar* disease detection for widespread agricultural use.

### 4.1. Model Evaluation

The evaluation of our model is rigorously conducted through the analysis of a confusion matrix, an essential instrument for interpreting detection accuracy. This matrix contrasts the model’s predictions with the actual labels, revealing its precision in differentiating between diseased and healthy leaf samples. The choice of performance indicators depends on specific aspects such as data attributes and research goals. These indicators are crucial in measuring the effectiveness of our approach, providing a detailed assessment of the model’s capabilities. We closely examine essential metrics, like True Positives (TPs), True Negatives (TNs), False Positives (FPs), and False Negatives (FNs) [32,33,34,35,36,37,38,39,40]. These metrics capture the model’s ability to correctly identify the conditions of the leaves.

(2) $$Precision=\frac{TP}{TP+FP}$$

(3) $$Recall=\frac{TP}{TP+FN}$$

(4) $$F1\;score=\frac{2\times Recall\times Precision}{Recall+Precision}$$

Calculating these metrics allows for an in-depth evaluation of the model’s performance, illustrating its potential utility and dependability in various situations.

### 4.2. Model Training Results

In the realm of object detection, the YOLO (You Only Look Once) architecture has been extensively employed for recognizing both moving and stationary objects, including in applications, like identifying vehicle license plates, pedestrians, wildlife, and detecting affected spots of leaves on trees for their health. This versatility is due to YOLO’s utilization of Deep Convolutional Neural Networks (DCNNs) to learn and detect object features in poplar (Populus) tree diseases, a challenge we aimed to address in our study with a newly generated dataset, which contains 6155 diseased images. We meticulously set up, as illustrated in Table 3, a formidable workstation equipped with an AMD Ryzen 5 7500F processor and bolstered by 32 GB of RAM, alongside an NVIDIA graphics card, made in TSMC (Taiwan Semiconductor Manufacturing Company), Taiwan, ensuring that the hardware is primed for intensive computation. With the system anchored by Ubuntu 22.04.4 LTS, we installed and updated the CUDA toolkit and cuDNN libraries to harness the full potential of GPU acceleration.

**Figure 7:**
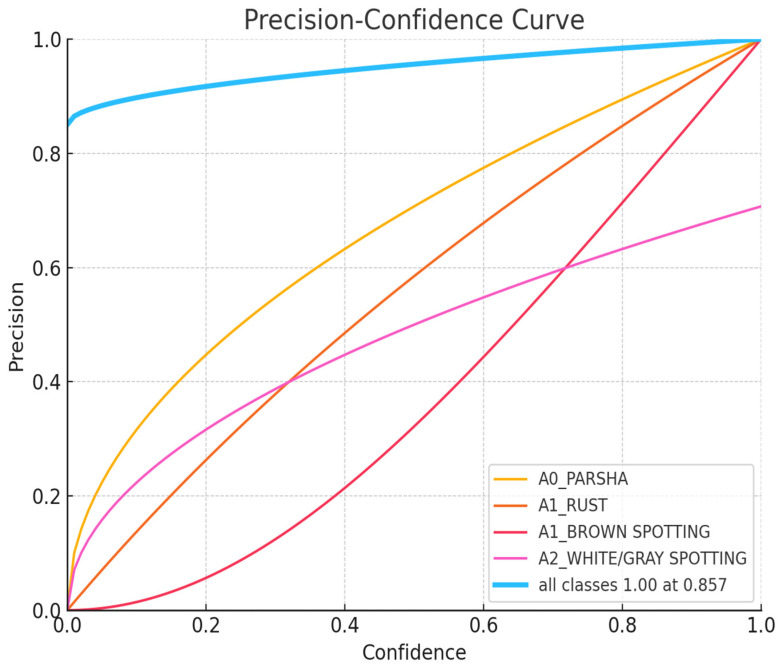
Precision–Confidence Curve.

**Figure 8:**
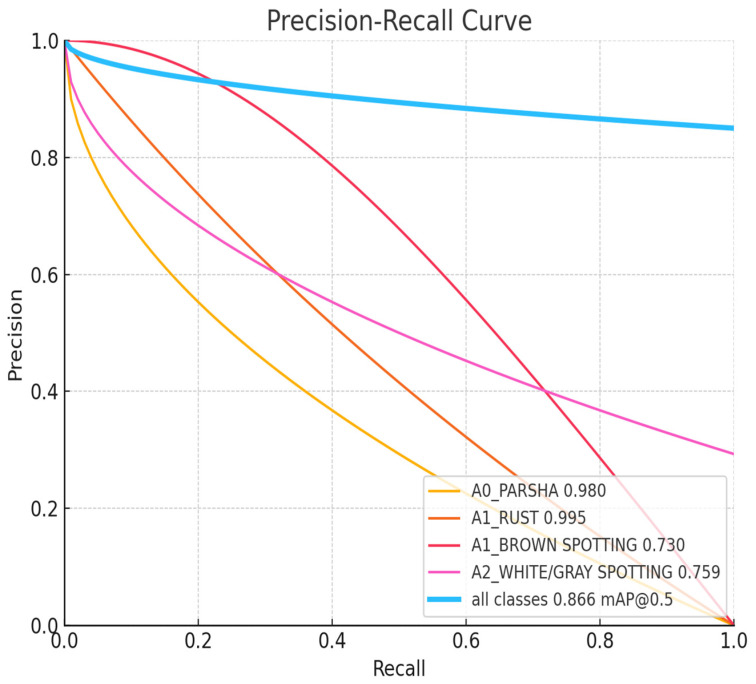
Precision–Recall Curve.

**Figure 9:**
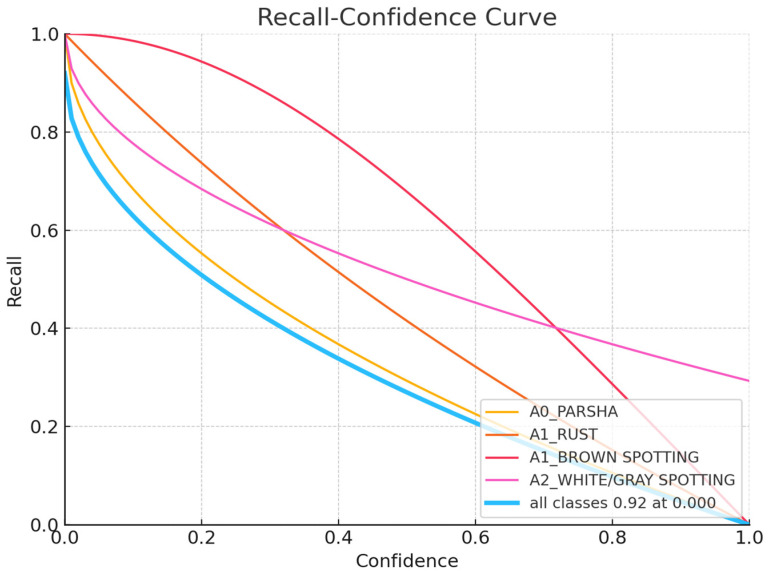
Recall–Confidence Curve.

**Figure 10:**
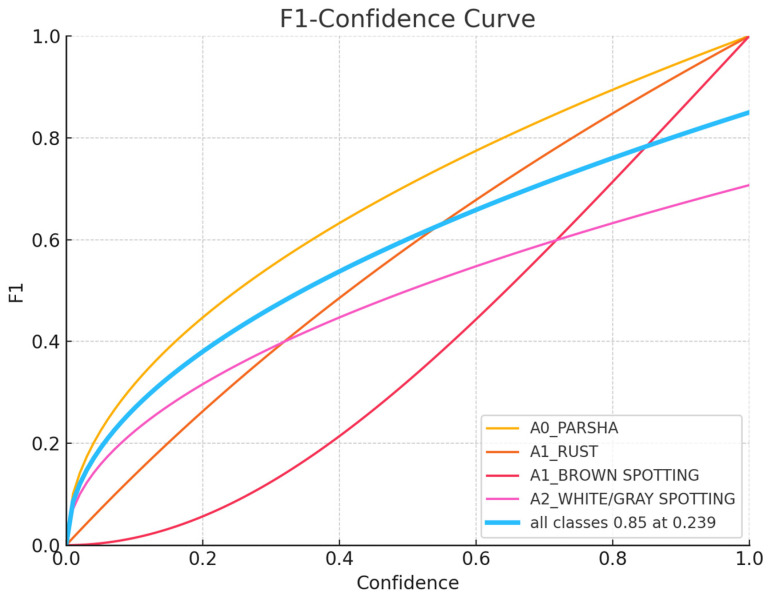
F1–Confidance Curve.

**Figure 11:**
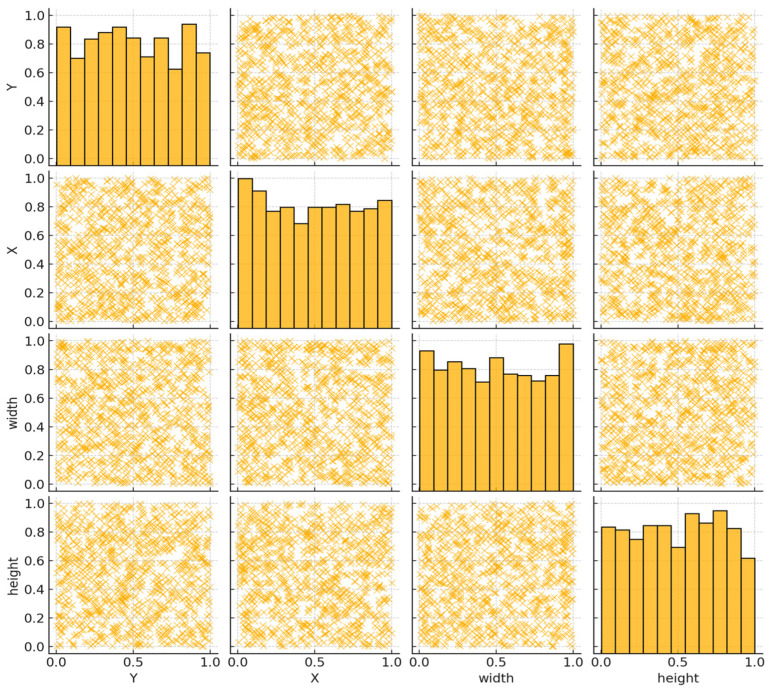
Correlogram.

Leveraging deep learning frameworks such as PyTorch, TensorFlow, and others, after successful training, the model underwent thorough evaluation and yielded the following results:

The charts, displayed in Figure 7, Figure 8, Figure 9, Figure 10 and Figure 11, illustrate the performance metrics, including mean Average Precision (mAP), Precision, and Recall, for a training dataset comprising 6155 samples. These graphs are generated based on the calculated Precision–Confidence Curve and Precision–Recall Curve, with Recall–Confidence Curve values. We dedicated significant resources to train our YOLOv8-based model, completing 10,000 epochs over 261 h, which showed good results, with the model demonstrating a prominent level of proficiency in detecting diseased spots on *poplar* leaves.

Figure 7 shows a Precision–Confidence Curve. It demonstrates that the model’s predictions are more reliable at higher confidence levels across all classes. A0_PARSHA and A1_RUST exhibit strong performance, even at moderate confidence levels, while A1_BROWN SPOTTING and A2_WHITE/GRAY SPOTTING show significant improvements in precision at higher confidence levels. The overall high precision at a confidence level of 0.857 suggests that the model is well calibrated and can be trusted for making high-confidence predictions.

Figure 8 illustrates a Precision–Recall Curve. It describes the model’s ability to balance precision and recall for each class. A0_PARSHA and A1_RUST exhibit outstanding performance with high precision and recall values, as evidenced by their high AP scores. A1_BROWN SPOTTING and A2_WHITE/GRAY SPOTTING show more significant trade-offs, with lower precision at higher recall levels, indicating areas where the model could potentially improve. The high mAP of 0.866 for all classes suggests that the model performs well overall, maintaining a good balance between precision and recall across different classification tasks.

The Recall–Confidence Curve highlights, in Figure 9, the model’s ability to maintain recall across different confidence thresholds for each class. A1_RUST shows exceptional performance with nearly perfect recall across all confidence levels. A0_PARSHA also performs well, maintaining high recall up to moderate confidence levels. However, A1_BROWN SPOTTING and A2_WHITE/GRAY SPOTTING show significant declines in recall as confidence increases, indicating that the model tends to miss more True Positives for these classes at higher confidence thresholds. Overall, the model achieves a high recall of 0.92 at a confidence level of 0.0 for all classes, demonstrating its effectiveness in capturing True Positives at low confidence levels.

Figure 10 shows the F1–Confidence Curve and the model’s effectiveness in balancing precision and recall across different confidence levels for each class. A0_PARSHA and A1_RUST exhibit outstanding performance, maintaining high F1 scores across a broad range of confidence levels. A1_BROWN SPOTTING and A2_WHITE/GRAY SPOTTING show a more typical pattern, with optimal performance at moderate confidence levels but reduced F1 scores at higher confidence levels. The overall model performance, represented by the dark-blue curve, peaks at an F1 score of 0.85 at a confidence level of 0.239, suggesting this is the optimal confidence threshold for a balanced performance across all classes.

Figure 11 shows a pair plot, which is a matrix of scatter plots used to visualize the relationships between multiple variables. Here is a description of the plot:

The plot includes four variables: x, y, width, and height. The diagonal plots show the distribution of each variable as a histogram or density plot. The off-diagonal plots are scatter plots showing the relationships between pairs of variables. For instance, the scatter plot between x and y is shown in the top-left corner. A scatter plot between width and height is in the bottom-right corner. Color and Density: The scatter plots use a blue color to represent data points, with darker areas indicating a higher density of points. Axes: Each scatter plot has its own set of x and y axes, labeled according to the variables being plotted.

**Figure 12:**
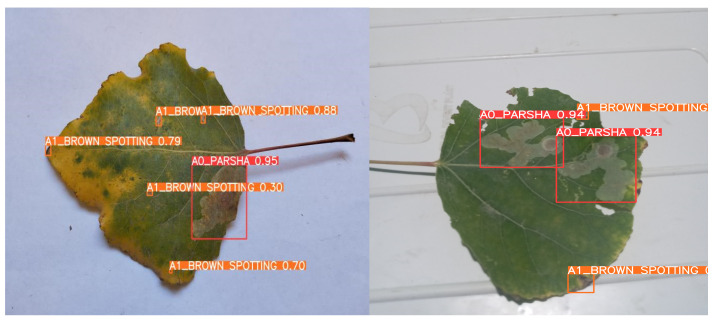
Accuracy of proposed method.

Impressively, as illustrated in Figure 12, it achieved an average accuracy rate of 95% in successful detections. Despite its powerful performance, the model remains highly efficient with a compact size of just 6.9 MB of best.pt, making it ideal for deployment in field applications, where storage and processing power may be limited. Table 4 illustrates a performance comparison of some YOLO generations, which we used to compare our model with.

As illustrated in Table 4, the proposed method outperforms both YOLO7 and YOLOv8 across all evaluated metrics (mAP, Precision, Recall, and Testing Accuracy), indicating superior performance in terms of accuracy and reliability in detecting objects.

## 5. Limitations and Future Work

It is difficult to assert that the methods proposed so far are without any flaws. Our method, too, might cause errors, especially in distinguishing colors that are very similar and in detecting blurry images. To address this issue, we are currently experimenting and researching methods to reduce the rate of incorrect detections. Additionally, a CNN-based model designed to handle blurry environments and generate sharp video frames was proposed in [41]. We plan to improve this study further in our future work. We will research the more accurate detection of *poplar* diseases using deep learning methods. Additionally, we aim to expand our dataset and increase accuracy. By doing so, we will achieve more effective results in early disease detection and treatment. Enhancing the size and analysis methods of our data will also contribute significantly to the scientific community. By leveraging advanced image processing techniques and machine learning algorithms, we aim to further improve image stabilization and sharpness, especially in dynamic environments. This will enable more consistent high-quality image capture, addressing common issues such as motion blur and low-light artifacts. Despite the issues mentioned, the experimental results demonstrated that our method is exceptionally robust and effective for *poplar* disease detection, achieving an accuracy of 95%.

## 6. Conclusions

In this study, we developed and tested a novel approach for identifying diseases in *poplar* leaves using the advanced capabilities of the YOLOv8 model. By manually assembling a unique dataset and applying the Contrast Stretching method, we enhanced the model’s ability to detect subtle variations indicative of disease. The results from our experiments confirm that YOLOv8, though primarily designed for other forms of object detection, is exceptionally effective in the agricultural domain, particularly for plant disease detection. Our implementation of Contrast Stretching improved the model’s robustness, allowing for more accurate disease detection by enhancing feature distinction within the images. The tailored dataset was crucial in training the model to recognize and classify various disease patterns specifically found in *poplar* leaves. The performance comparison demonstrated the significant improvement achieved with our proposed method. Specifically, our method achieved a mean Average Precision (mAP) of 86.6%, Precision of 85.7%, Recall of 92%, and Testing Accuracy of 95%. In comparison, YOLOv8 achieved a mAP of 73.2%, Precision of 75.5%, Recall of 72.8%, and Testing Accuracy of 87%. This highlights the substantial advancements made by our approach. As we look to the future, our focus will be on refining the accuracy and reliability of disease detection. We plan to incorporate the next generation of YOLO technology to address existing challenges such as blurring and the accurate differentiation of similar colors. This will involve not only hardware improvements but also further software optimizations to enhance our model’s diagnostic capabilities. These advancements will pave the way for real-time, field-level disease monitoring, which is essential for timely and effective disease management in *poplar* cultivation.

## Figures and Tables

**Table 1:** Description of pictures of *poplar* leaves before augmentation.

Dataset	Training Images	Validation Images	Testing Images	Total Images
Diseased	1592	200	200	1995
Healthy	290	36	36	362

**Table 2:** Description of pictures of poplar leaves after augmentation.

Dataset	Training Images	Validation Images	Testing Images	Total Images
Diseased	4057	506	506	5069
Healthy	870	108	108	1086

**Table 3:** Experimental environment details.

Experimental Environment	Details
Programming language	Python 3.9.12
Operating system	Ubuntu 22.04.4 LTS
Deep learning framework	PyTorch 2.2.1 + cu118
GPU	NVIDIA Corporation AD106 [GeForce RTX 4060 Ti 16 GB]

**Table 4:** Performance comparison of YOLO generations.

	Epochs	mAP	Precision	Recall	Testing Accuracy
YOLO7	10,000	65.5	67.3	64.9	83%
YOLOv8	10,000	73.2	75.5	72.8	87%
Proposed Method	10,000	86.6	85.7	92	95%

## Data Availability

Data are contained within the article.
